# Publisher Correction: Organosilicon uptake by biological membranes

**DOI:** 10.1038/s42003-021-02344-2

**Published:** 2021-06-23

**Authors:** Pepijn Beekman, Agustin Enciso-Martinez, Sidharam P. Pujari, Leon W. M. M. Terstappen, Han Zuilhof, Séverine Le Gac, Cees Otto

**Affiliations:** 1 Applied Microfluidics for BioEngineering Research, MESA+ Institute for Nanotechnology & TechMed Center, University of Twente, Enschede, The Netherlands; 2 Laboratory of Organic Chemistry, Wageningen University, Wageningen, The Netherlands; 3 Medical Cell BioPhysics, TechMed Center, University of Twente, Enschede, The Netherlands; 4 School of Pharmaceutical Science and Technology, Tianjin University, Tianjin, China; 5 Department of Chemical and Materials Engineering, Faculty of Engineering, King Abdulaziz University, Jeddah, Saudi Arabia

**Keywords:** Biophysical chemistry, Nanoscale biophysics

Correction to: *Communications Biology* 10.1038/s42003-021-02155-5, published online 9 June 2021.

In the original version of this Article, the legends to Figures 1, 2, 3, and 4 were inadvertently swapped. This has now been corrected in the PDF and HTML versions of the Article.

