# Author Correction: Organosilicon uptake by biological membranes

**DOI:** 10.1038/s42003-021-02338-0

**Published:** 2021-06-23

**Authors:** Pepijn Beekman, Agustin Enciso-Martinez, Sidharam P. Pujari, Leon W. M. M. Terstappen, Han Zuilhof, Séverine Le Gac, Cees Otto

**Affiliations:** 1 Applied Microfluidics for BioEngineering Research, MESA+ Institute for Nanotechnology & TechMed Center, University of Twente, Enschede, The Netherlands; 2 Laboratory of Organic Chemistry, Wageningen University, Wageningen, The Netherlands; 3 Medical Cell BioPhysics, TechMed Center, University of Twente, Enschede, The Netherlands; 4 School of Pharmaceutical Science and Technology, Tianjin University, Tianjin, China; 5 Department of Chemical and Materials Engineering, Faculty of Engineering, King Abdulaziz University, Jeddah, Saudi Arabia

**Keywords:** Biophysical chemistry, Nanoscale biophysics

Correction to: *Communications Biology* 10.1038/s42003-021-02155-5, published online 9 June 2021.

The original version of this Article contained an error in the spelling of the author Han Zuilhof, which was incorrectly given as Han T. Zuilhof. This has now been corrected in both the PDF and HTML versions of the Article.

