# Supplementary material for: Organosilicon uptake by biological membranes
Source: Commun Biol. 2021 Jun 9;4:704. doi: 10.1038/s42003-021-02155-5 (PMC8190035; doi:10.1038/s42003-021-02155-5)
Supplement: Supplementary file 2 — Supplemental Material [file 42003_2021_2155_MOESM2_ESM.pdf]

Supporting Information

SI1 structures

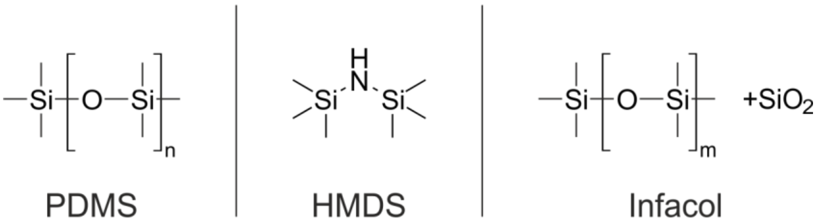

Figure SI1. Structures of the molecules used in this study. For uncrosslinked PDMS oligomers, n = 20-90. m = 190-280.

SI2 AES

Figure SI2. AES data

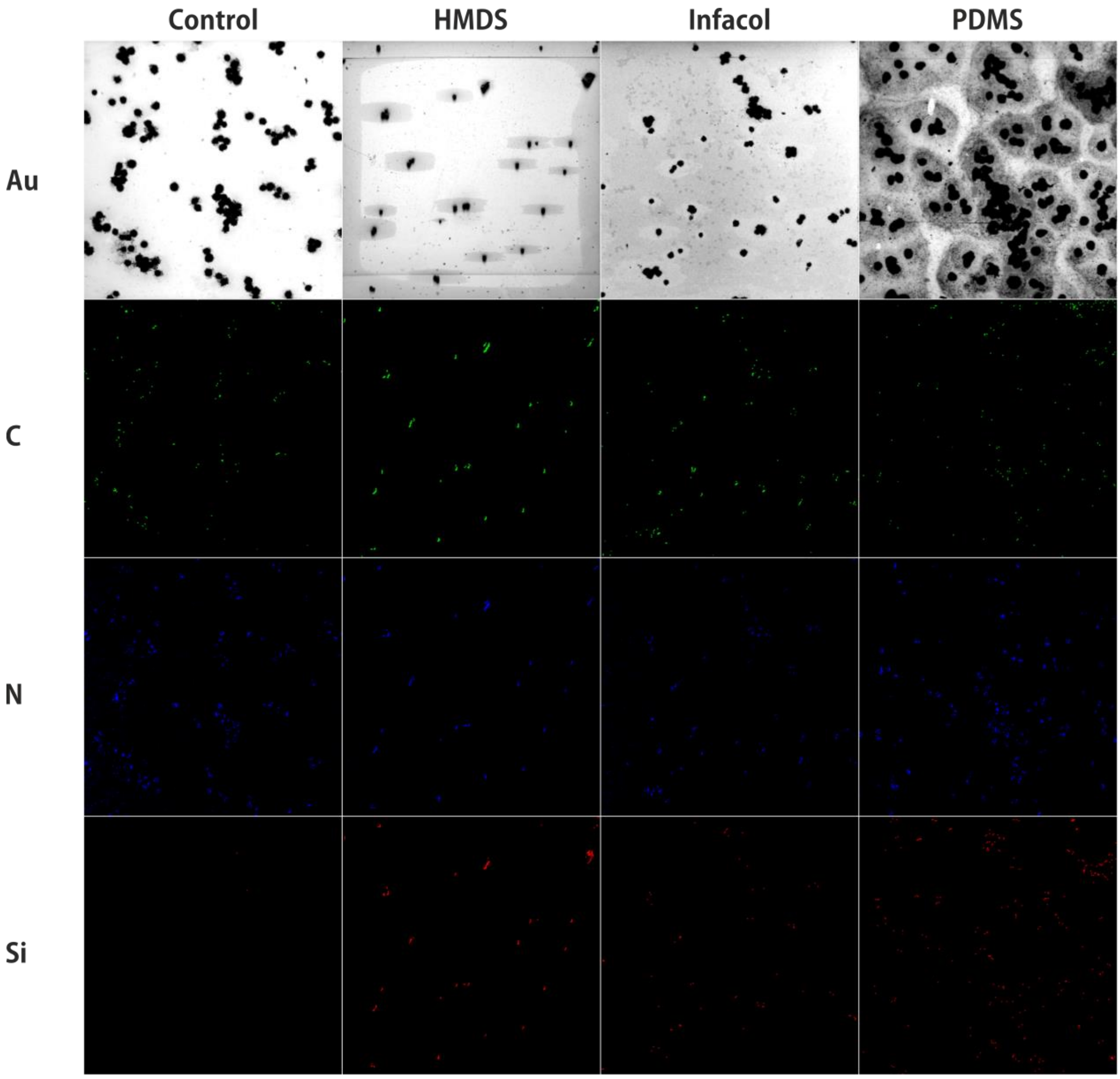

Nitrogen species are observed in all cells and an intense gold signal arises from the substrate, for comparison. (C and Si data also shown in main article after image dilution).

SI3 IR

Figure SI3. Full IR spectra.

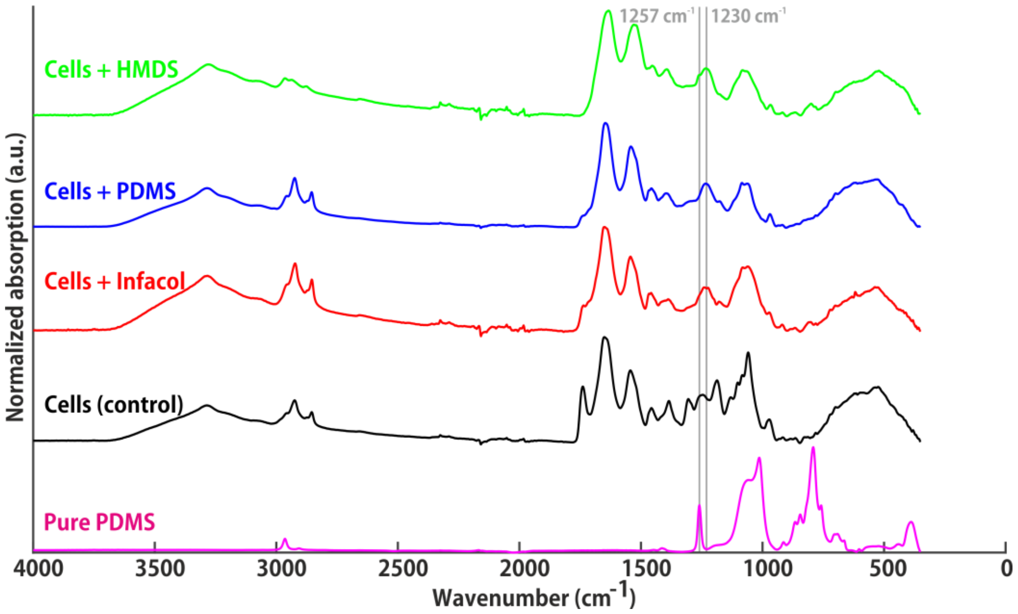

The region of interest, 1180 – 1300 cm^{-1}, is shown in the main text.

SI4 Raman Spectroscopy

Figure SI4. Raman spectra.

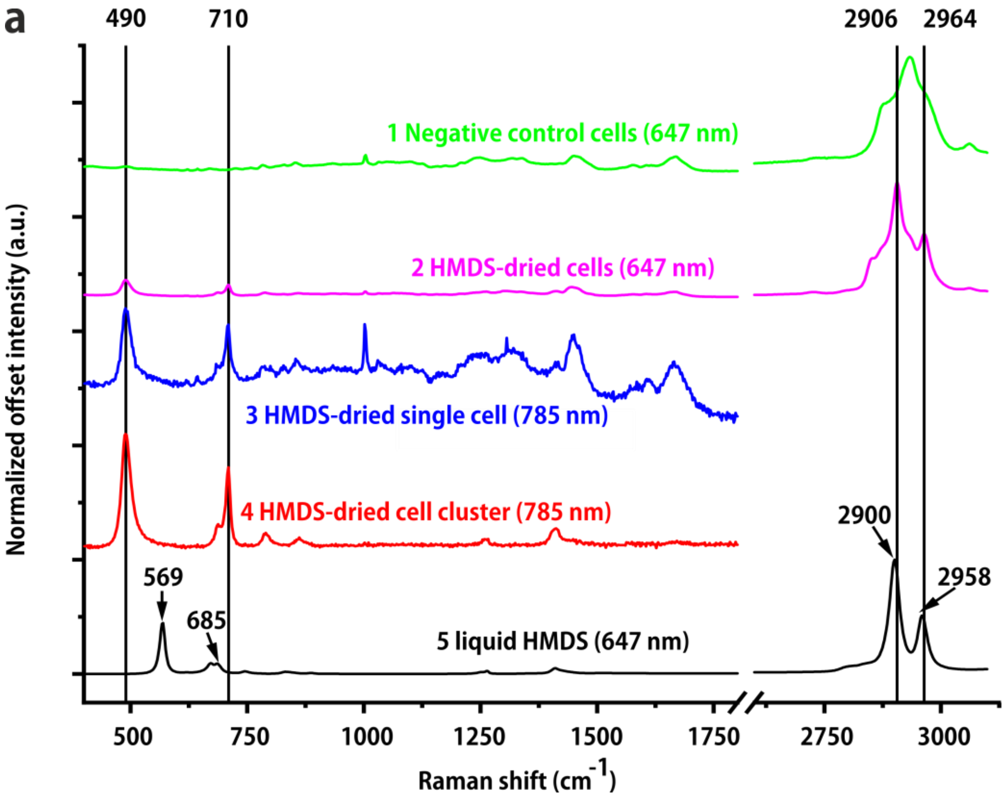

Raman spectra (3) and (4) correspond to a cell dried with HMDS (3) and to a cluster of dried cells with excess HMDS (4). Both, (3) and (4) display peaks at 490 and 710 cm^{-1}. The cell contribution to the Raman spectrum in (4) is weak compared to the peaks at 490 and 710 cm^{-1}, indicating that the interaction of HMDS with biological matter is efficient and can dominate the response in Raman imaging.
