# Supplementary material for: Organosilicon uptake by biological membranes
Source: Commun Biol. 2021 Jun 9;4:704. doi: 10.1038/s42003-021-02155-5 (PMC8190035; doi:10.1038/s42003-021-02155-5)
Supplement: Supplementary file 3 — Description of Additional Supplementary Files [file 42003_2021_2155_MOESM3_ESM.pdf]

Description of Additional Supplementary Files

File name: Supplementary Data 1

Description: Data to Figure 2: a) Raman spectra, b) Raman images, c) AES-SEM images

File name: Supplementary Data 2

Description: Data to Figure 3: a) XPS spectra, b) XPS zoom in, c) IR spectra
